# Commensal Bacteria-Dependent Indole Production Enhances Epithelial Barrier Function in the Colon

**DOI:** 10.1371/journal.pone.0080604

**Published:** 2013-11-20

**Authors:** Yosuke Shimada, Makoto Kinoshita, Kazuo Harada, Masafumi Mizutani, Kazunori Masahata, Hisako Kayama, Kiyoshi Takeda

**Affiliations:** 1 Laboratory of Immune Regulation, Department of Microbiology and Immunology, Graduate School of Medicine, Osaka University, Osaka, Japan; 2 Laboratory of Mucosal Immunology, WPI Immunology Frontier Research Center, Osaka University, Osaka, Japan; 3 Core Research for Evolution Science and Technology, Japan Science and Technology Agency, Saitama, Japan; 4 Applied Environmental Biology, Graduate School of Pharmaceutical Sciences, Osaka University, Osaka, Japan; 5 Morishita Jintan Co., Ltd., Osaka Techno Center, Osaka, Japan; Charité, Campus Benjamin Franklin, Germany

## Abstract

Microbiota have been shown to have a great influence on functions of intestinal epithelial cells (ECs). The role of indole as a quorum-sensing (QS) molecule mediating intercellular signals in bacteria has been well appreciated. However, it remains unknown whether indole has beneficial effects on maintaining intestinal barriers *in vivo*. In this study, we analyzed the effect of indole on ECs using a germ free (GF) mouse model. GF mice showed decreased expression of junctional complex molecules in colonic ECs. The feces of specific pathogen-free (SPF) mice contained a high amount of indole; however the amount was significantly decreased in the feces of GF mice by 27-fold. Oral administration of indole-containing capsules resulted in increased expression of both tight junction (TJ)- and adherens junction (AJ)-associated molecules in colonic ECs in GF mice. In accordance with the increased expression of these junctional complex molecules, GF mice given indole-containing capsules showed higher resistance to dextran sodium sulfate (DSS)-induced colitis. A similar protective effect of indole on DSS-induced epithelial damage was also observed in mice bred in SPF conditions. These findings highlight the beneficial role of indole in establishing an epithelial barrier *in vivo*.

## Introduction

The human gastrointestinal tract contains trillions of microorganisms, called commensal gut microbiota. Commensal microbiota establish a symbiotic relationship with their host, to maintain homeostasis of the gut environment. For example, gut commensal microbiota support host metabolism by producing energy and nutrients from the diet [1]–[3]. Furthermore, recent reports demonstrate that gut microbiota influence not only metabolic processes but also the development of the host immune system and the maintenance of the intestinal mucosal barrier [4], [5].

Various mechanisms by which gut microbiota modulate epithelial functions were recently reported [6], [7]. Gut microbiota can influence the functions of epithelial cells (ECs) either through the direct contact with ECs, or indirectly through mediating the production of dietary metabolites. The direct recognition of microbiota by ECs is mainly mediated by Toll-like receptors (TLRs), which comprise a family of pattern recognition receptors. TLR2 promotes the assembly of intestinal epithelial tight junction (TJ)-associated molecules via phosphatidylinositol 3-kinase (PI3K) and protein kinase B (Akt) pathway [8]. TLR4 regulates proliferation and apoptosis of ECs [9]. Indirect effect by microbiota can occur through a variety of mechanisms. For example, acetic acid produced by a certain bifidobacterial strain, one of the major bacterial species in probiotics, was recently shown to promote the defense function of host ECs against enterohemorrhagic *Escherichia coli* (EHEC) by inducing genes related to anti-inflammatory and anti-apoptosis effects of ECs [10]. Short-chain fatty acids (SCFAs) fermented by intestinal microbiota were also reported to activate G-protein-coupled receptors such as GPR41 and GPR43 expressed in ECs, leading to inflammatory cytokine production [11]. Thus, microbiota directly or indirectly affect epithelial barrier functions by modulating various pathways in ECs.

Just as mammalian cells use a variety of cytokines and hormones to communicate with each other, microbes also utilize specific molecules for transducing certain signals to others, which are called quorum-sensing (QS) molecules. Acyl-homoserine lactones (AHLs) are one of the best-characterized classes of molecules involved in this process [12]. The effects of AHLs on bacterial cells include toxin expression, regulation of virulence, and cell growth. A previous report showed that gut commensal microbes also utilize this QS system for communication [13]. More importantly, some of these QS molecules were recently shown to modulate immune responses in the host. The *Pseudomonas sp.* QS molecule, 3-oxododecanoyl homoserine lactone (3-oxo-C_12_-HSL) induced apoptosis of macrophages and neutrophils [14]. Thus, certain bacterial QS molecules mediate communication between bacteria and their host [15], [16].

Indole is produced by a variety of both gram-positive and gram-negative bacteria possessing tryptophanase, a bacteria-specific enzyme that catabolizes tryptophan. Indole has been shown to act as a QS molecule that mediates intercellular signals in bacteria [17]. A recent study indicated that indole enhances barrier functions of ECs *in vitro* by inducing the expression of several genes involved in EC functions [18]. These genes included those responsible for TJs, adherens junctions (AJ), actin cytoskeleton and mucin production, indicating the role of indole in strengthening the epithelial barrier. However, it remains unknown whether indole has beneficial effects on maintaining the intestinal barrier *in vivo*.

In this study, we showed that colonic ECs expressed decreased level of TJ- and AJ-associated molecules in GF mice. Compared to the feces of SPF mice, the feces of GF mice contained significantly less amount of indole. Oral administration of indole-containing capsules resulted in enhanced expression of both TJ- and AJ-associated molecules in colonic ECs of GF mice. Furthermore, beneficial effects of indole against DSS-mediated epithelial insult were observed not only in GF mice but in SPF mice. These results suggest that indole produced by gut commensal microbiota plays an essential role in enhancing epithelial barrier functions in the colon.

## Materials and Methods

### Reagents

Indole was purchased from Sigma, and 3-indoxyl sulfate potassium salt was purchased from Alfa Aesar. N,N-Dimethylformamide (DMF) was purchased from Nacalai Tesque. Indole was dissolved in medium chain triglycerides (MCT), and seamless microcapsules containing either indole or MCT were prepared at Morishita Jintan. Anti-occludin rabbit polyclonal IgG was purchased from Invitrogen. Anti-E-cadherin mouse polyclonal IgG was purchased from BD Transduction Laboratories. Alexa Fluor 568-conjugated anti-rabbit IgG, and Alexa Fluor 568-conjugated anti-mouse IgG were purchased from Life Technologies. 4, 6-diamidino-2-phenylindole (DAPI) was purchased from Wako.

### Mice

ICR and IQI mice were purchased from CLEA Japan. ICR mice are a well-appreciated outbred strain used as SPF mice, whereas IQI mice are GF mice developed from the ICR strain in Japan. IQI mice were bred and maintained in vinyl isolators under GF conditions. For the administration of indole or MCT, mice were given indole- or MCT- containing seamless microcapsules (approximately 15 mg) once daily for 2 weeks by oral catheters. Fifteen mg of microcapsules contained 0.369 mg of indole. All animal experiments were performed following our institutional guidelines.

### Sample preparation for HPLC analysis

After feces were collected into microtubes, a 10-fold volume of methanol was added and the feces were homogenized. Two hundred microliters of the suspension was transferred to a new tube, and 200 µL of methanol was added. The mixture was then incubated at −20°C for 1 h, and centrifuged at 20,000× g for 15 min at 4°C. Subsequently, 150 µL of the supernatant was collected and 150 µL of distilled water was added. The solution was centrifuged at 20,000× g for 15 min at 4°C and the supernatant was applied to HPLC analysis.

### HPLC analysis for indole

HPLC analyses were performed with a HITACHI L-2000 (Hitachi High-Technologies, Tokyo, Japan). Separations were carried out at 30°C with an Inertsil ODS-3, 5 µm, 4.6×250 mm (GL sciences). Solvent A was 0.1% (v/v) formic acid, and solvent B was acetonitrile. The initial composition of the binary solvent was B 50% from 0 to 5.0 min. Solvent B was increased from 50 to 100% over 5.0 min. The composition of solvent remained for 5.0 min at B 100%, with the flow rate set at 1.0 mL min^−1^. Ten microliters of the sample solution was subjected and fluorescence was monitored. The excitation and emission wavelengths were 280 and 335 nm respectively. The sampling rate was set at 0.4 second. The data at one run was acquired for 20 min. The control of instrument, data acquisition, and data analysis were performed with a D-2000 Elite (Hitachi High-Technologies).

### Sample preparation for LC/MS/MS analysis

Blood was drawn from the heart using a heparinized syringe, and centrifuged at 3,000× g for 10 min at 4°C. Two hundred microliters of methanol and 50 µL of 0.4 µM 4-methylumbelliferyl sulfate (internal standard, in 15% acetonitrile) were added to 50 µL of the collected serum, and then the sample was incubated at −20°C for 1 h. Subsequently, the solution was centrifuged at 20,000× g for 15 min at 4°C and the supernatant was dried in a vacuum centrifugal dryer. Afterward, the residue was dissolved with 100 µL of 15% acetonitrile. The solution was centrifuged at 20,000× g for 5 min at 4°C. The supernatant was applied to LC/MS/MS analysis.

### LC/MS/MS analysis for indoxyl sulfate

LC/MS/MS analyses were performed on a Waters ACQUITY UPLC system (Waters) coupled to a Qattro Premier XE triple quadrupole mass spectrometer (Waters). LC separations were carried out at 30°C with an Acquity UPLC BEH C18 column, 1.7 µm, 2.1×100 mm (Waters). Solvent A was 0.1% (v/v) formic acid, and solvent B was acetonitrile. The initial composition of the binary solvent was 15% B from 0 to 3.0 min. Solvent B was increased from 15 to 100% over 2.0 min and the composition of solvent remained for 1.0 min at 100% B. The flow rate was set at 0.3 mL min^−1^. Five microliters of sample solution was applied to LC/MS/MS analysis. Mass spectrometer was operated using an electrospray ionization source in the negative mode. The ionization parameters were capillary voltage, 4.5 kV; extractor voltage, 2 V; source temperature, 120°C; desolvation temperature, 350°C; desolvation gas flow, 800 L/h; cone gas flow, 50 L/h. Selected reaction monitoring (SRM) was conducted. SRM transitions (*m/z* of precursor ion/*m/z* of product ion) for indoxyl sulfate were 212.0/79.9 (quantification) and 212.0/131.8 (identification). For a former transition, cone voltage and collision energy were set at 26 V and 22 eV. For a latter transition, they were set at 26 V and 18 eV. SRM transitions for 4-methylumbelliferyl sulfate were 255.0/174.9 (quantification) and 255.0/132.8 (identification). For the former transition, cone voltage and collision energy were set at 28 V and 16 eV, while for the latter transition, they were set at 28 V and 36 eV. The dwell time for each SRM transition was set at 100 ms and the data at one run were acquired for 10 min. The control of instrument, data acquisition, and data analysis were performed with a MassLynx 4.1 (Waters).

### Isolation of intestinal epithelium

Intestines were incised longitudinally, washed to remove fecal content, and incubated in HBSS containing 5 mM EDTA for 20 min at 37°C in a shaker, followed by vortexing for 1 min. After centrifugation at 2000 rpm for 20 min at 4°C, the pellet was used as the intestinal epithelium.

### Real-time RT-PCR

Epithelial samples were collected separately from 4 individual mice maintained under either SPF or GF condition. Four µg of RNA was reverse transcribed using M-MLV reverse transcriptase (Promega) and random primers (Toyobo) after treatment with RQ1 DNase I (Promega). cDNAs were analyzed by qPCR using the GoTaq qPCR Master Mix (Promega) in ABI 7300 real-time PCR system (Applied Biosystems). All data were normalized to the expression of *Gapdh*, and the fold difference in expression relative to that of *Gapdh* is shown. Amplification conditions were: 50°C (2 min), 95°C (10 min), 40 cycles of 95°C (15 s), and 60°C (60 s). Primers of *Gapdh, Cldn7, Ocln, Tjp1, Ctnnb1, Cdh1* were purchased from Invitrogen. The sequences of primers are listed in Table S1.

### Caco-2 cell culture

Approximately 1×10^4^ Caco-2 cells were cultured on transwell filters of 3.0 µm in the pore size (BD Biosciences). When the transepithelial electrical resistance (TEER) reached 1000 Ω (World Precision Instruments ENDOHM 6), cells were considered to have become confluent. After confirming the full confluency, 1 mM indole or 2 mM indoxyl sulfate was added to both the upper and lower chambers. DMF or PBS was used as controls respectively. After 24 h incubation under each condition, total RNA was extracted from the cultured cells by TRIzol reagent.

### Induction of DSS colitis

Colitis was induced in male mice at the age of 8- to 12- weeks by adding DSS (M.W = 36000–50000; MP Biomedicals) in the drinking water for 5 days. DSS containing water was replaced by tap water after 5 days, and mice were given the water until the end of the experiments. Both body weights and survival rates in each group were monitored throughout the experiments. The final concentration of the DSS in the drinking water varied from 4 to 5% (w/v) as indicated in each experiment.

### Immunohistochemistry

Mouse intestinal tissues were fixed with 4% paraformaldehyde (Wako), and immersed in 30% sucrose for 24 h. The fixed tissues were embedded in OCT compound (Sakura), and sections were prepared with a thickness of 10 µm. Sections were blocked by 1% BSA in PBS with Tween 20 (PBS-T) for 30 min, and stained with either anti-occludin or anti-E-cadherin (1∶100) antibody in PBS-T containing 0.1% BSA for 24 h at 4°C, followed by secondary antibodies for 1 h at room temperature. Sections were stained with DAPI for staining the nucleus, and mounted with PermaFluor (Thermo SCIENTIFIC). Images were captured using a confocal microscope (FV1000-D; Olympus).

### Statistical analysis

One-way ANOVA and unpaired student's t-test were used to determine statistical significance. P values of less than 0.05 were considered significant.

## Results

### Expression levels of junctional complex molecules are decreased in colonic epithelia of GF mice

**Figure 1:**
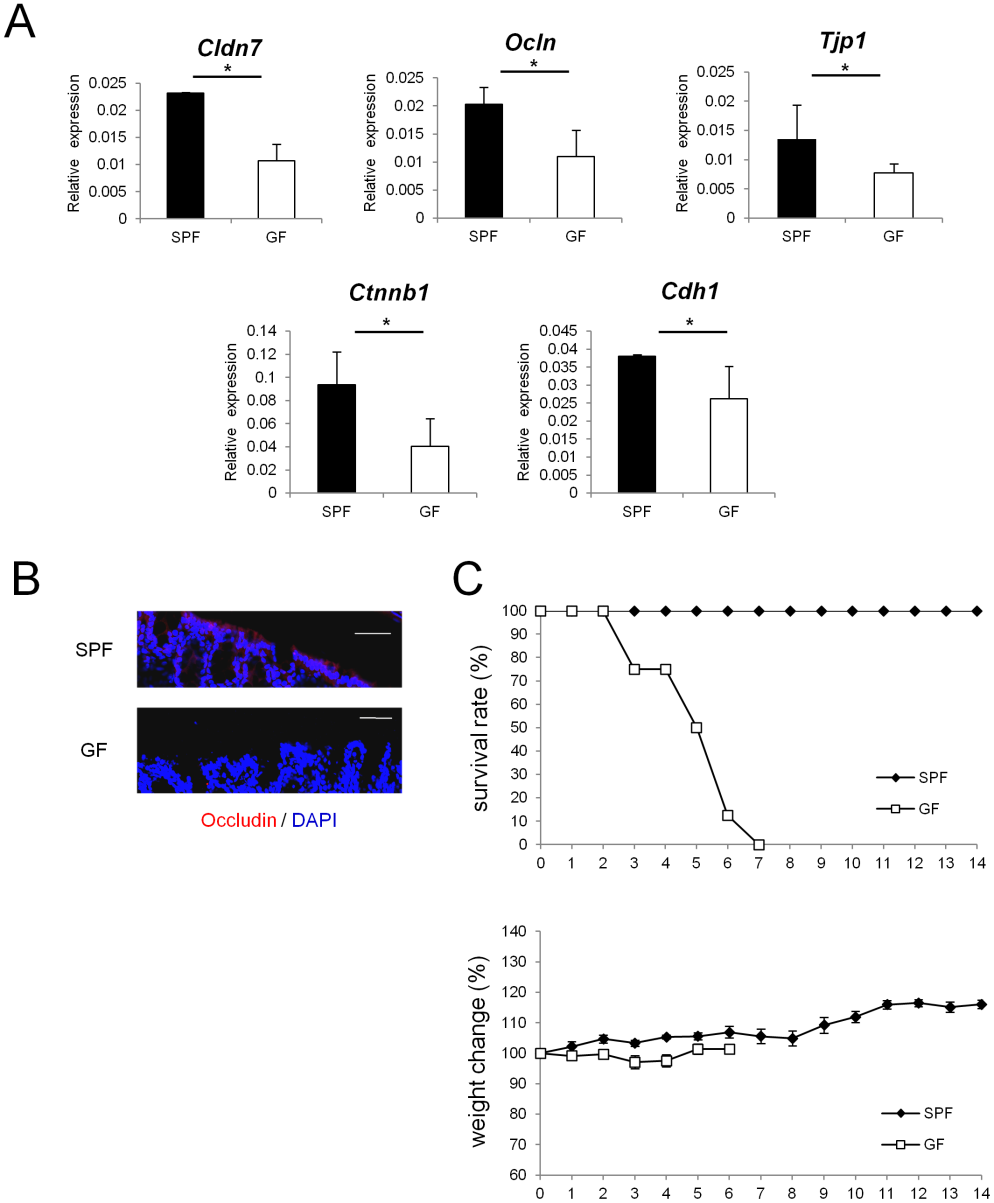
Epithelial barrier functions is impaired in GF mice. (A) Real-time quantitative RT-PCR analysis of mRNA expression of *Cldn7, Ocln, Tjp1, Ctnnb1, Cdh1* in colonic ECs in SPF (n = 4) or GF (n = 4) mice. Values were normalized to that of *Gapdh*. Data are representative of two independent experiments and show mean values ± S.D. of 4 samples performed in duplicate. *P<0.05. (B) Mouse colonic tissue was stained with anti-occludin antibody. Sections were analyzed using a confocal microscope. Bars, 50 µm. Data are representative of two independent experiments. (C) SPF (n = 8) or GF (n = 8) mice were administered 4% DSS by drinking water for 3 days. Survival rates of the indicated mice are shown. Body weight changes relative to the value prior to colitis induction are shown. Data are mean ± S.E.M of 8 mice at each time point. SPF, specific pathogen free; GF, germ free.

To examine whether commensal microbiota play an essential role in establishing the epithelial barrier in the gut, mRNA expression of TJ- or AJ-associated molecules were analyzed in SPF and GF mice. Expression of TJ-associated molecules, such as *Cldn7*, *Ocln*, and *Tjp1*, which encode claudin-7, occludin, and zonula occludens (ZO)-1, respectively, was lower in the colonic epithelia of GF mice than in those of SPF mice (Figure 1A). In addition, expression of mRNAs that encode AJ-associated molecules, represented by *Ctnnb1* (encoding β-catenin) and *Cdh1* (encoding E-cadherin), was also lower in GF mice than in SPF mice (Figure 1A). In contrast, none of the TJ- or AJ-associated molecules that were shown to be decreased in the colonic epithelia of GF mice were decreased in the small intestines of GF mice (Figure S1).

Immunohistochemical analysis further demonstrated that in GF mice, protein expression of occludin was lower than in SPF mice (Figure 1B). These results suggest that in the absence of commensal microbiota, expression of junctional complex molecules in colonic ECs is reduced. To examine whether the decrease in the expression of junctional complex molecules affects susceptibility to chemical insult of gut epithelium, we challenged SPF and GF mice with oral DSS treatment. SPF mice and GF mice were treated with 4% DSS for 3 days, and the survival rates and changes in weight were monitored. In accordance with a previous report [19], GF mice were more sensitive to DSS-induced epithelial damage compared with SPF mice (Figure 1C).

### Indole concentration is decreased in the feces of GF mice

**Figure 2:**
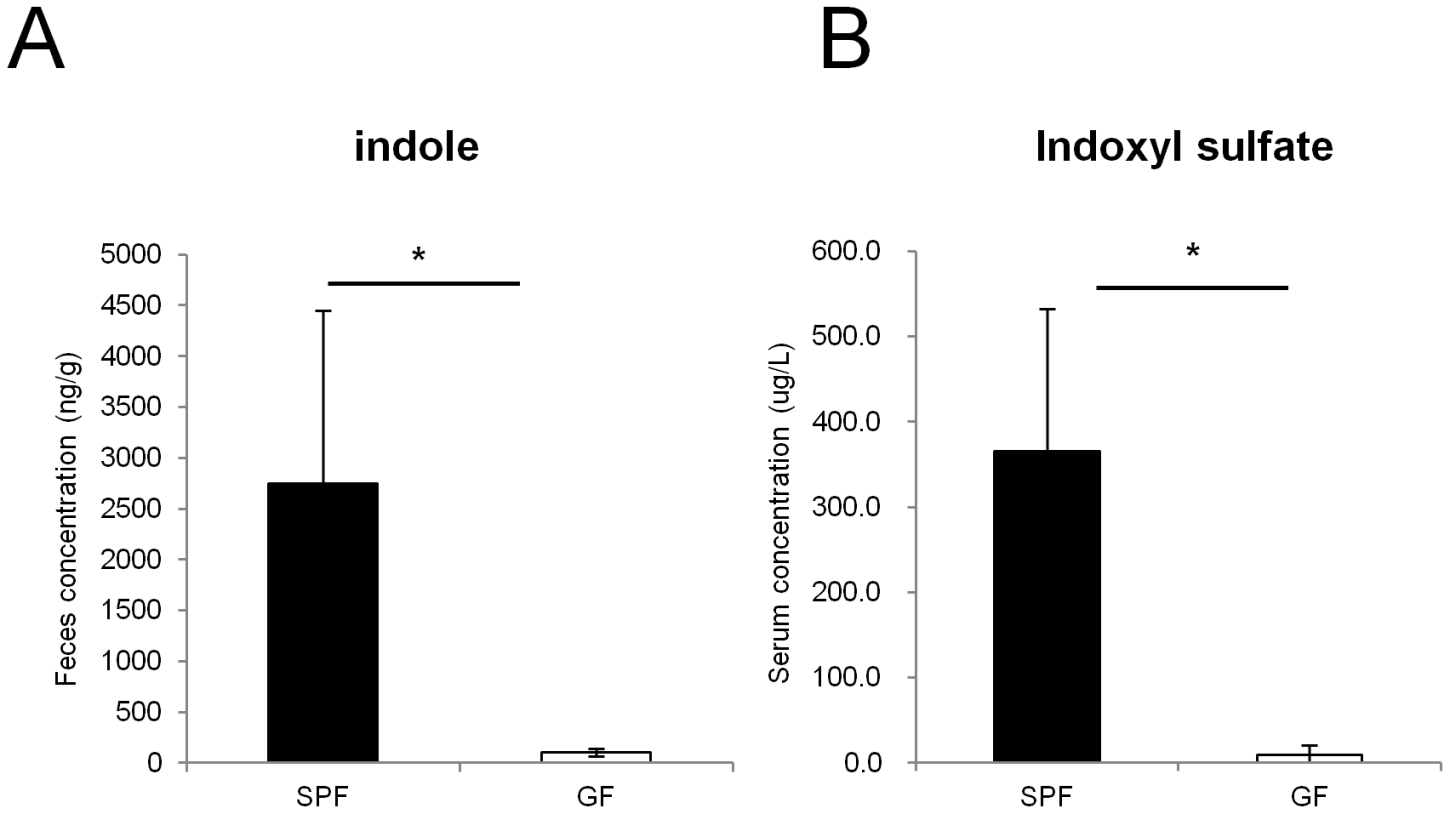
Indole and indole metabolites are absent in GF mice. (A, B) Feces and serum were collected from either SPF (n = 3) or GF (n = 3) mice. The concentration of indole in the feces was measured by HPLC-FL, and the serum concentration of indoxyl sulfate was measured by LC-MS/MS. Data are representative of two independent experiments and show mean values ± S.D. of 3 mice. *P<0.05. SPF, specific pathogen free; GF, germ free.

**Figure 3:**
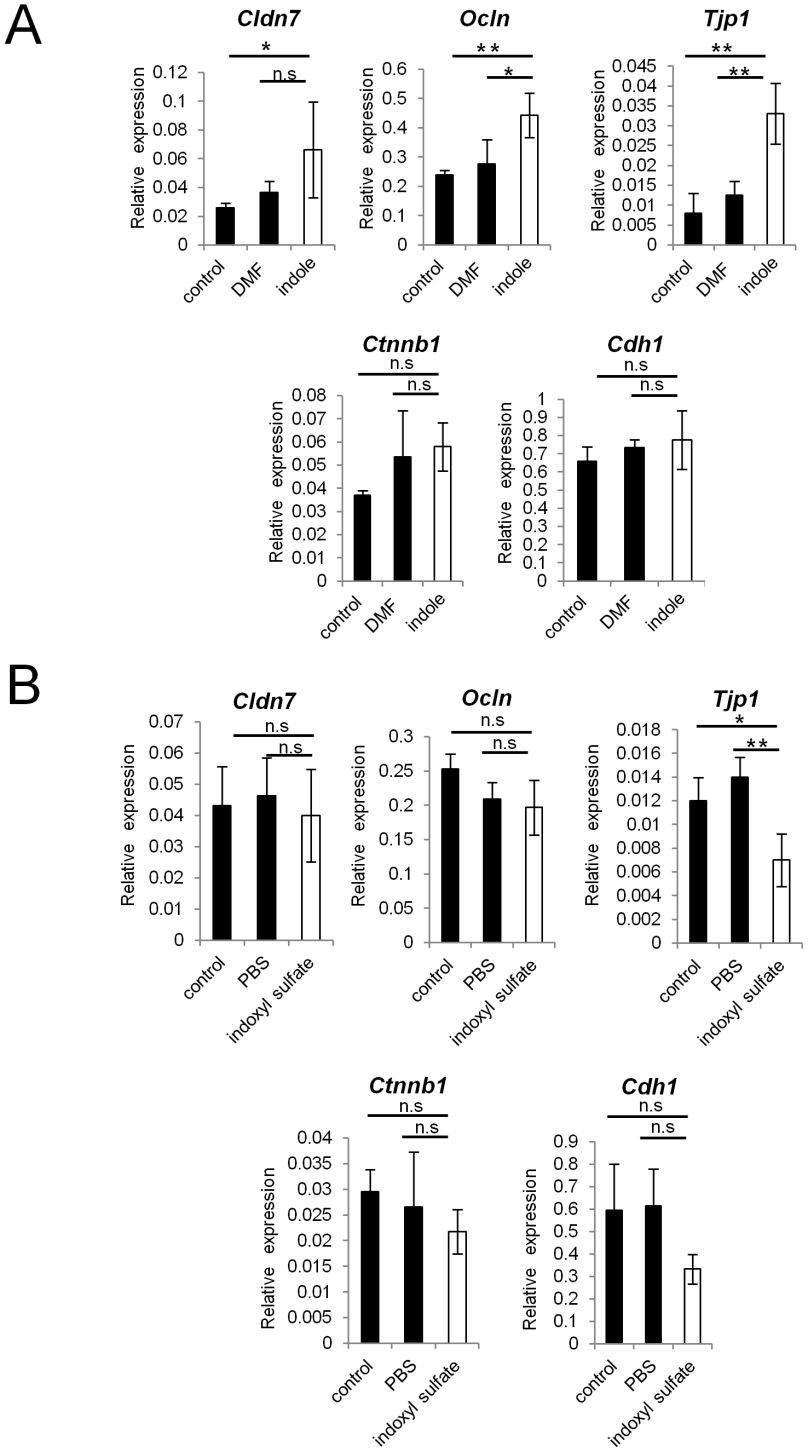
Indole, but not indoxyl sulfate, induces the expression of junctional complex molecules in Caco-2 cells. (A, B) Real-time quantitative RT-PCR analysis of mRNA expression of *Cldn7, Ocln, Tjp1, Ctnnb1, Cdh1* in Caco-2 cells cultured with indole or indoxyl sulfate is shown. DMF or PBS was used as a control, respectively. Quadruplicate was used for each condition. Values were normalized to the expression of *Gapdh*. Data are representative of two independent experiments and show mean values ± S.D. of 4 samples performed in duplicate. *P<0.05. n.s., not significant.

We next examined which factor produced by commensal microbiota enhanced the barrier function of the colonic epithelial cells. Indole was previously reported to enhance the expression of various genes related to junctional complexes in the human enterocyte cell line, HCT-8 [18]. Therefore, to analyze the extent to which commensal microbiota contribute to the production of indole in the intestinal lumen, we measured the concentration of indole in the feces of SPF mice and GF mice by an HPLC-FL assay (Figure 2A). Indole concentration was severely reduced in the feces of GF mice compared with those of SPF mice by 27-fold. When the host absorbs indole, it is metabolized to indoxyl sulfate by specific enzymes [20], [21]. Indeed, LC-MS/MS analysis showed that serum concentration of indoxyl sulfate was severely decreased in GF mice (Figure 2B). Thus, commensal microbiota contribute to production of substantial amount of indole in the gut lumen, and to the increase in the concentration of indole metabolites. We next analyzed whether indole or indoxyl sulfate play a role in establishing intestinal epithelial barrier. Indole promoted mRNA expression of TJ-associated molecules, such as *Cldn7, Ocln,* and *Tjp1* in the human adenocarcinoma cell line, Caco-2 (Figure 3A). This is consistent with previously reported observations in HCT-8 cells [18]. In contrast, indoxyl sulfate-treatment rather showed a slight reduction in the mRNA expression levels of all of these genes (Figure 3B). The mRNA expression of AJ-associated molecules, represented by *Ctnnb1* and *Cdh1*, was unaffected in both indole- and indoxyl sulfate-treated conditions. Thus, in Caco-2 cells, expression of TJ-associated molecules is induced by indole, but not its metabolite, indoxyl sulfate.

### GF mice given indole-containing capsules show enhanced expression of junctional complex molecules, and are more resistant to DSS-induced epithelial damage

**Figure 4:**
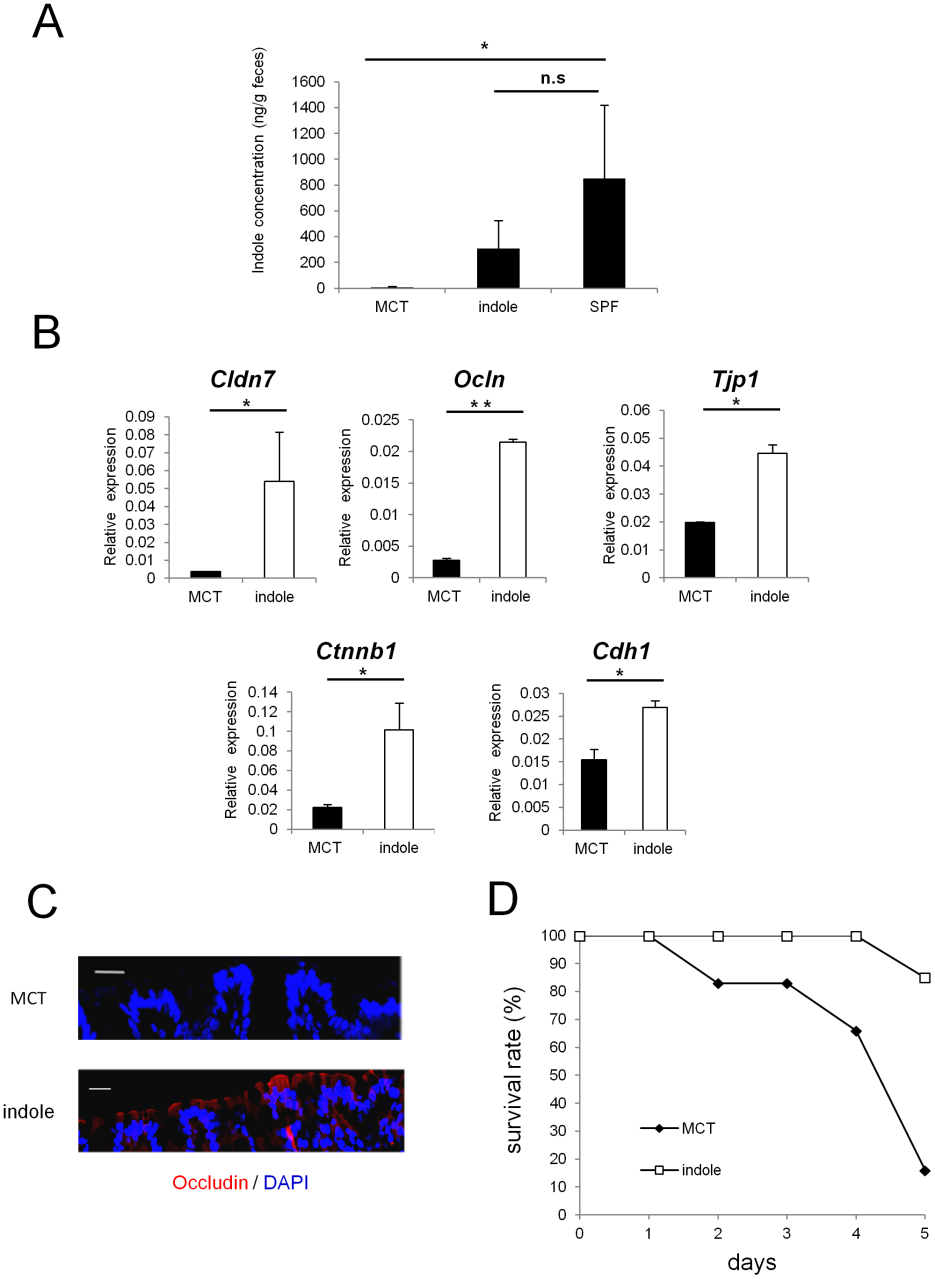
Indole-containing capsules promote epithelial barrier function in GF mice. (A) Feces were collected from SPF mice, and GF mice treated with indole- or MCT- containing capsules. Three mice was analysed in each group. The concentration of indole in the feces was measured by HPLC-FL. Data show mean values ± S.D. of 3 samples. *P<0.05. n.s., not significant. SPF, specific pathogen free; GF, germ free; MCT, Medium-Chain Triglycerides. (B) Real-time quantitative RT-PCR analysis of mRNA expression of *Cldn7, Ocln, Tjp1, Ctnnb1*, and *Cdh1* in colonic epithelial cells of GF mice treated with indole- (n = 4) or MCT- (n = 4) containing capsules. Values were normalized to the expression of *Gapdh*. Data are representative of two independent experiments and show mean values ± S.D. of 4 samples performed in duplicate. *P<0.05. (C) Colonic tissues of GF mice treated with indole- or MCT- containing capsules were stained with anti-occludin antibody. Sections were analyzed using a confocal microscope. Bars, 20 µm. Data are representative of two independent experiments. (D) After oral administration with either indole- (n = 6) or MCT- (n = 6) containing capsules for 2 weeks, GF mice were treated by 4% DSS in drinking water for 3 days. Survival rate of the mice in each group is shown. Data are representative of two independent experiments. MCT, Medium-Chain Triglycerides.

To examine whether indole possesses the capacity to enhance intestinal epithelial barrier functions *in vivo*, we used seamless microcapsules to deliver the compound to the colonic epithelium [22]. To confirm the successful delivery and the dissolution of these capsules at the colon, capsules containing carbon powder were prepared and administered orally to the mice. Three hours after the administration, the capsules were shown to successfully dissolve at the end portion of the small intestine (Figure S2). MCT- or indole- containing capsules were then prepared following the same manufacturing processes. HPLC analysis showed that indole concentration in the feces of GF mice given indole-containg capsules for 2 weeks reached approximately one third of that observed in SPF mice (Figure 4A). These mice given indole-containing capsules for 2 weeks showed increased mRNA expression of *Cldn7*, *Ocln*, and *Tjp1* in colonic ECs. Additionally, mRNA expression of AJ-associated molecules, such as *Ctnnb1* and *Cdh1*, was also increased in GF mice given indole-containing capsules (Figure 4B). In contrast, none of the junctional complex molecules that showed increased expression in the colonic ECs were increased in the small intestine, reflecting the release of the active compound from the capsules in the distal small intestine (Figure S3).

Immunohistochemical analysis further demonstrated the increase in occludin expression in the colonic epithelia for the indole-treated group (Figure 4C). Because indole was suggested to play a critical role in establishing epithelial junctional complexes, we next examined whether indole treatment ameliorates the disease course in GF mice challenged with DSS. After indole- or MCT-containing capsules were given orally for 2 weeks, GF mice were given 4% DSS, and survival rates were monitored. Only 15% of GF mice treated with indole-containing capsules died during the induction of DSS-colitis, whereas the mortality rate was over 90% in the MCT-treated group (Figure 4D).

### Indole reduces the weight loss of SPF mice with DSS-induced colitis

**Figure 5:**
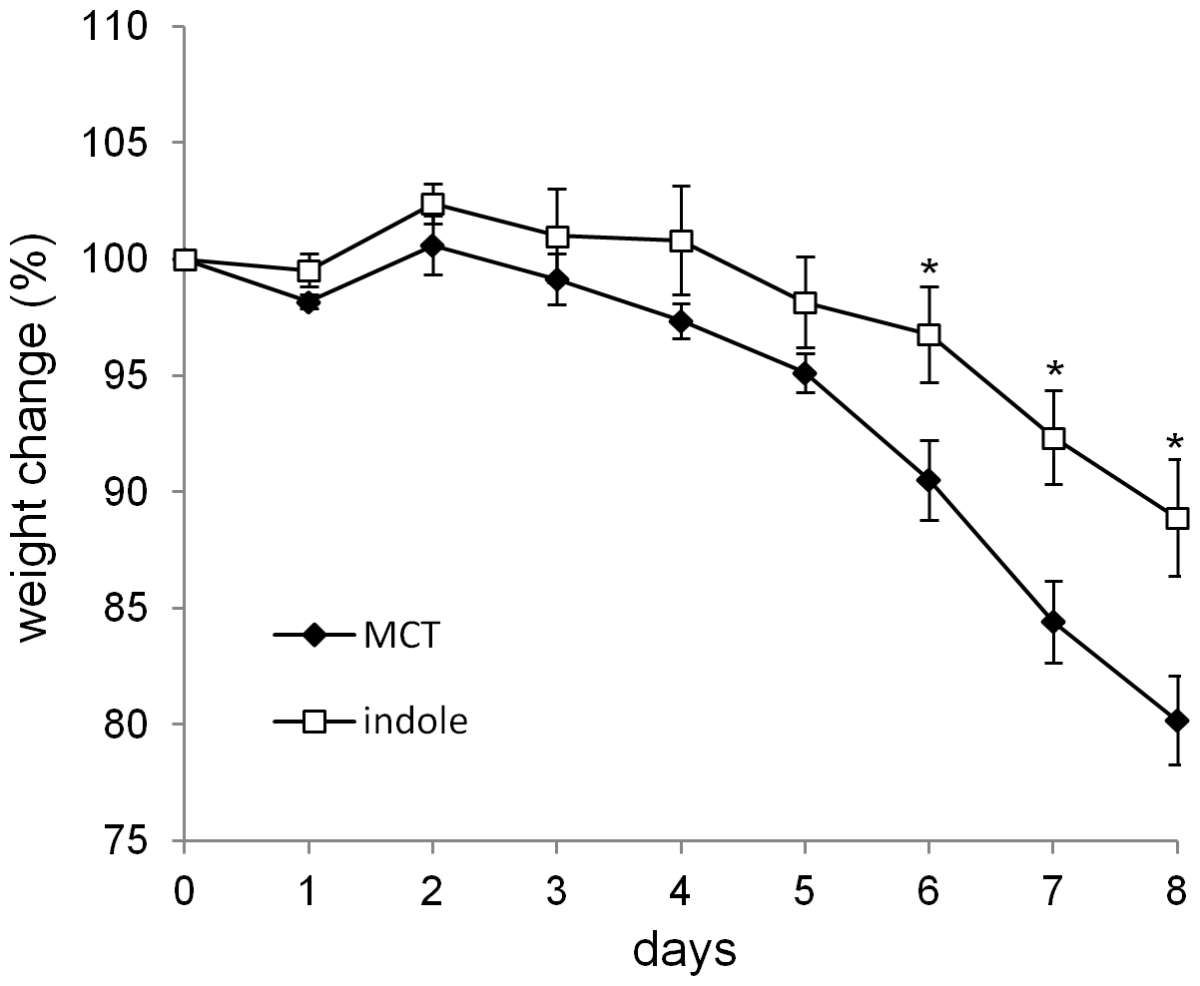
Indole-containing capsules show preventative effect on colitis development in SPF mice. SPF mice were treated with indole- (n = 7) or MCT- (n = 7) containing capsules for 1 week, and then challenged by 5% DSS for 6 days. Body weight changes relative to the value prior to colitis induction are shown. Data are representative of two independent experiments and mean ± S.E.M of 7 mice at each time point is shown. *P<0.05. MCT, Medium-Chain Triglycerides.

Because indole was shown to have beneficial effects against DSS-mediated epithelial damage in GF mice, we next examined whether indole could also ameliorate the disease course in SPF mice. When SPF mice were treated with either indole- or MCT-containing capsules for 1 week prior to the induction of DSS colitis, significant decrease in the weight loss was observed in the indole-treated group (Figure 5). Thus, indole treatment has beneficial effects on DSS-mediated epithelial impairment, even in the presence of physiological level of indole.

## Discussion

In the current study, we demonstrated that indole, a bacterial QS molecule, promotes the establishment of the intestinal epithelial barrier *in vivo*. Expression of junctional complex molecules was decreased in colonic epithelia of GF mice, where indole production was lower as measured from feces. GF mice given indole-containing capsules showed increased expression of TJ- and AJ-associated molecules. GF mice treated with indole-containing capsules showed a higher resistance to epithelial damage induced by DSS. The preventative effect of indole against DSS was also demonstrated in SPF mice.

Our study demonstrated that the gut epithelium of GF mice had significantly reduced expression of TJ- and AJ-associated molecules exclusively in the colon. Considering that as much as 10^11–13^ bacteria exist per gram of colonic intestinal content [23], the presence of commensal microbiota is suggested to be crucial for the proper development of the colonic intestinal barrier. Indeed, several reports demonstrated that certain strains of commensal bacteria possess the capacity to promote intestinal barrier integrity. *Escherichia coli* strain Nissle 1917, for example, was shown to promote the expression and redistribution of ZO-2 *in vitro* [24]. *Bacteroides thetaiotaomicron* was reported to prevent TEER decrease in cell monolayers after treatment with TNF-α and IFN-γ [25]. Our study showed that indole produced by commensal microbiota contributes to this process. In addition to indole, there are several QS molecules used by microbes such as 7-hydroxyindole, isatin, and competence and sporulation factor (CSF) [26], [27]. CSF derived from *Bacillus subtilis*, for example, was reported to activate p38 and Akt pathway, and also induces heat shock proteins, which prevent oxidant-induced impairment in ECs [28]. Identifying other QS molecules with essential roles in maintaining the intestinal epithelial barrier is warranted in the future investigations.

Unlike in the colon, in the small intestine of GF mice, there was no significant alteration in the expression of junctional complex molecules. A previous study showed that extracts of certain foods, such as linden and star anise, decrease the permeability of Caco-2 cell monolayers [29]. Deprivation of glutamine was also reported to result in reduced claudin-1 expression, and TEER decrease in Caco-2 cells [30]. Given that the main function of the small intestine is absorption of dietary nutrition, the induction of junctional complexes in the small intestine might be regulated by certain dietary components. Characterizing the dietary substances that promote epithelial barrier functions will help us understand the regulatory mechanism of the mucosal barrier in the small intestine.

Both our *in vitro* and *in vivo* studies clearly demonstrated that indole treatment induced the mRNA expression of junctional complex molecules. This is consistent with the previous report that showed indole enhanced the TJ-associated molecule mRNAs, such as *Cldn7* and *Tjp1*, in HCT-8 cells [18]. However, it remains unknown which specific receptor or signaling pathway is involved in indole-mediated regulation of host ECs. For the induction of occuldin, a direct interaction of thyroid transcripton factor-1 with the *Ocln* promoter is considerd to be essential [31]. The AP-1 transcription factor, JunD, was shown to regulate the transcription and translation of *Tjp1* [32]. On the other hand, previous report showed that the expression of *Cldn7* was regulated by ELF3, an epithelia-specific member of Ets family of transcriptional factors [33]. It would be interesting to investigate which of these pathways is specifically involved in the enhancement of the epithelial barrier by indole.

After indole is absorbed in the intestine, it is metabolized in phase 1 of xenobiotic metabolism to indoxyl by the cytochrome P450 isoform, Cyp2e1, then to indoxyl sulfate by sulfotransferase 1a1, Sult1a1, in phase 2. The conversion of indole into indoxyl sulfate is thought to occur in the liver [20], [21], but Cyp2e1 and Sult1a1 are expressed in colonic ECs [34], [35]. Therefore, it is possible that effects of indole on host ECs are mediated by one of its metabolites, indoxyl sulfate. Our *in vitro* studies, however, demonstrated that indole, but not its metabolite, indoxyl sulfate, serves as the critical factor to enhance the epithelial barrier functions. The fact that indole itself promotes epithelial barrier functions indicates that receptors that recognize hydrophobic ligands are somehow involved in the pathway by which this occurs [36]. It would be interesting to analyze whether other indole derivatives, such as hydroxyindole, which is known to serve as a QS molecule, are capable of exerting similar effects on epithelial barrier functions in the future.

In GF mice treated with indole, a higher resistance to DSS-mediated epithelial insult was observed. Previous studies have shown that breakdown of the mucous and epithelial barrier underlies the DSS-induced damage [37], [38]. In this regard, the importance of junctional complex molecules is suggested for the protection against DSS-induced epithelial damage [39]. In the acute phase of DSS-mediated colitis, junctional complexes are initially disrupted by DSS and this impairment allows the luminal bacteria to invade into the lamina propria, resulting in inflammatory responses at the chronic phase. The critical role of claudin-7 in preventing intestinal inflammation was previously reported. Mice deficient for *Cldn7* were reported to suffer from spontaneous development of colitis [40]. Thus, indole-mediated up-regulation of TJ-associated molecules might contribute to the resistance to intestinal inflammation. Alternatively, the observed changes in the tight junction proteins may reflect an increase in epithelial polarity. Whether the protection conferred by indole-treatment is mediated by enhanced mucous layer by these well-differentiated ECs would be an interesting issue to investigate in the future.

It is of note that the beneficial effects of indole were also observed in SPF mice challenged with DSS. This observation suggests that oral supplementation of indole can enhance the mucosal barrier functions even in the condition when the physiological level of indole is present. In recent years, probiotics have attracted much attention for their preventative effect on intestinal inflammation [41]. It would be interesting to examine whether commensal microbiota-derived metabolites can be applied in the future as a therapeutic option for the treatment of patients with inflammatory bowel disease.

## Supporting Information

Figure S1 The mRNA expression levels of various molecules related to TJ and AJ in the epithelium of small intestines in GF mice. Real-time quantitative RT-PCR analysis of mRNA expression of *Cldn7, Ocln, Tjp1, Ctnnb1, Cdh1* in the epithelium of small intestines in SPF (n = 4) or GF (n = 4) mice. Values were normalized to that of *Gapdh*. Data are representative of two independent experiments and show mean values ± S.D. of 4 samples performed in duplicate. *P<0.05. n.s., not significant. SPF, specific pathogen free; GF, germ free.
(TIF)

Figure S2 Carbon-containing seamless capsules dissolve at the end portion of small intestines after the administration by oral route. To confirm the delivery system of seamless capsules at the end portion of small intestines, mice were given carbon-containing microcapsules (approximately 15 mg) by oral catheters. After 3 h, intestines were incised longitudinally.
(TIF)

Figure S3 The mRNA expression of TJ- and AJ-associated molecules in the small intestines of GF mice given indole-containing capsules. Real-time quantitative RT-PCR analysis of mRNA expression of *Cldn7, Ocln, Tjp1, Ctnnb1, Cdh1* in the epithelium of small intestines in GF mice treated with MCT-containing capsules (n = 4) and indole-containing capsules (n = 4). The values were normalized to that of *Gapdh*. Data are representative of two independent experiments and show mean values ± S.D. of 4 samples performed in duplicate. *P<0.05. n.s., not significant. MCT, Medium-Chain Triglycerides.
(TIF)

Table S1 Primers list used in this study.
(XLSX)
