# Supplementary figures and images for: Commensal Bacteria-Dependent Indole Production Enhances Epithelial Barrier Function in the Colon
Source: PLoS One. 2013 Nov 20;8(11):e80604. doi: 10.1371/journal.pone.0080604 (PMC3835565; doi:10.1371/journal.pone.0080604)

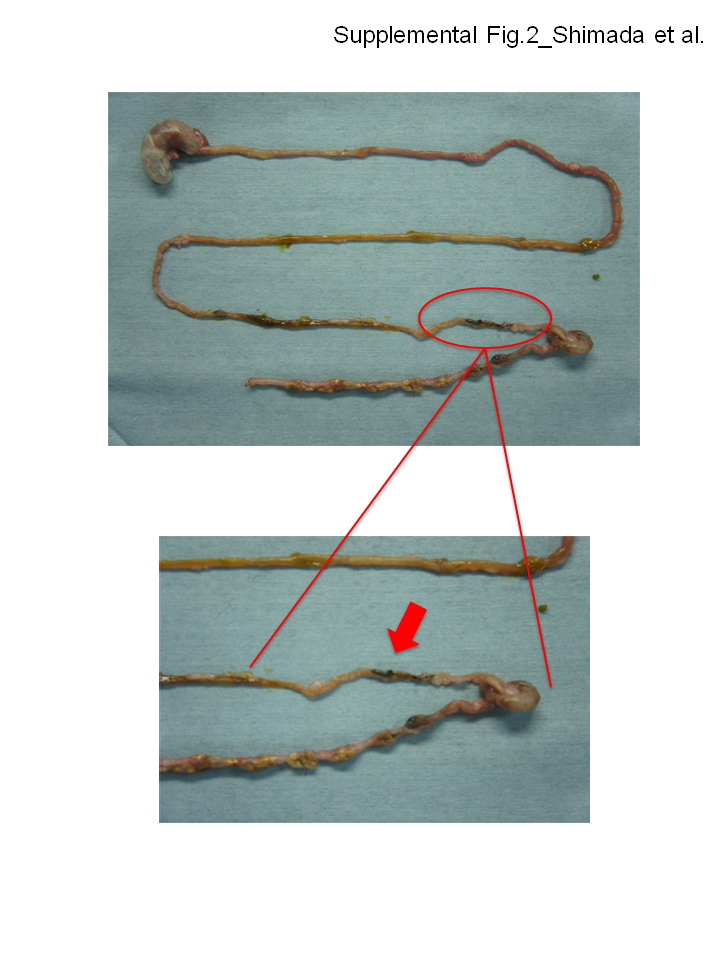

Supplement: Figure S2 — Carbon-containing seamless capsules dissolve at the end portion of small intestines after the administration by oral route. To confirm the delivery system of seamless capsules at the end portion of small intestines, mice were given carbon-containing microcapsules (approximately 15 mg) by oral catheters. After 3 h, intestines were incised longitudinally. (TIF) [file pone.0080604.s002.tif]
